# Development of Orodispersible Tablets with Solid Dispersions of Fenofibrate and Co-Processed Mesoporous Silica for Improved Dissolution

**DOI:** 10.3390/pharmaceutics16081060

**Published:** 2024-08-12

**Authors:** Ana Baumgartner, Odon Planinšek

**Affiliations:** Faculty of Pharmacy, University of Ljubljana, Aškerčeva 7, 1000 Ljubljana, Slovenia; ana.baumgartner@ffa.uni-lj.si

**Keywords:** co-processed excipient, fenofibrate, amorphous solid dispersion, orodispersible tablets, direct compression, dissolution enhancement

## Abstract

Poor water solubility is an important challenge in the development of oral patient-friendly solid dosage forms. This study aimed to prepare orodispersible tablets with solid dispersions of a poorly water-soluble drug fenofibrate and a co-processed excipient consisting of mesoporous silica and isomalt. This co-processed excipient, developed in a previous study, exhibited improved flow and compression properties compared to pure silica while maintaining a high specific surface area for drug adsorption. Rotary evaporation was used to formulate solid dispersions with different amounts of fenofibrate, which were evaluated for solid state properties and drug release. The solid dispersion with 30% fenofibrate showed no signs of crystallinity and had a significantly improved dissolution rate, making it the optimal sample for formulation or orodispersible tablets. The aim was to produce tablets with minimal amounts of additional excipients while achieving a drug release profile similar to the uncompressed solid dispersion. The compressed formulations met the requirements for orodispersible tablets in terms of disintegration time, and the drug release from best formulation approximated the profile of uncompressed solid dispersion. Future research should focus on reducing the disintegration time and tablet size to enhance patient acceptability further.

## 1. Introduction

Poor aqueous solubility of many pharmaceutical compounds represents an important challenge in the development of solid dosage forms intended for oral use. It has been estimated that as much as 90% of newly discovered compounds are poorly water-soluble and belong to either Class II or Class IV according to the Biopharmaceutical Classification System (BCS) [1,2]. Preparation of amorphous solid dispersions (SDs) has been recognized as one of the promising approaches to enhance the apparent solubility and dissolution rate of such compounds, because the amorphous state exhibits a disordered structure and possesses higher free energy [3]. In recent years, there has been an increasing research focus on mesoporous materials, especially mesoporous silica materials, as carriers for SDs. Due to their high surface area and high pore volume, the drug can adsorb inside the pores in an amorphous state and spreads over a large surface, which makes it available for subsequent dissolution in the aqueous environment [4]. This concept has already been proven successful on many model drugs and the detailed mechanism for it has also been thoroughly discussed [5].

However, the use of such SDs to produce final dosage forms is often limited by their poor powder flow and compression properties, making them inappropriate for capsule filling or tableting. To overcome these challenges, high proportions of excipients such as fillers, binders and disintegrants are needed to prepare a suitable powder blend [6]. Consequently, the mass and size of a final dosage form have to be relatively high to ensure sufficient drug content, which is undesired by patients, especially children and the elderly. As these patient groups are particularly challenging regarding patient compliance, there have been many incentives to promote the development of more patient-friendly dosage forms [7]. While dosage forms such as granules, pellets and mini-tablets are easier to swallow due to their small size, (oro)dispersible and effervescent dosage forms are produced and supplied as a solid, but ingested as a liquid dispersion [8]. Among the latter, orodispersible tablets (ODTs) are especially convenient, since they disintegrate rapidly in the oral cavity upon contact with the saliva, which makes them considered as the first choice for patients with swallowing dysfunction or dysphagia [9].

**Figure 1:**
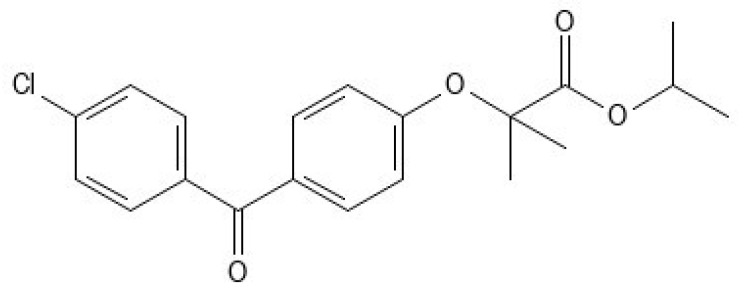
Fenofibrate.

Direct compression is the most common method to produce ODTs due to its ease of use and a greater cost effectiveness compared to methods such as lyophilization, molding, etc. [10]. However, since poor compressibility of the tablet ingredients can limit its applicability, one must choose the excipients wisely. One possibility is to include co-processed excipients, which are defined as a combination of two or more excipients designed to physically alter their properties in a manner not achievable by physical mixing, and without any significant chemical changes [11]. Co-processed excipients, when used in direct compression, can have a role of a filler and/or binder and can improve powder flow properties of a tableting blend. In some cases, they can even include a superdissintegrant, thereby being functionalized especially for ODTs [12]. In our previous study, we developed a co-processed excipient consisting of mesoporous silica and a sugar alcohol isomalt, which is appropriate for direct compression and has a sufficiently large specific surface area to make it suitable for drug loading and formulation of amorphous SDs [4]. In this study, our objective was to prepare SDs by loading a model BCS Class II drug fenofibrate (FF, Figure 1; log P = 5.24, aqueous solubility less than 0.1 mg/L, T_g_ < −20 °C) [13,14] onto this excipient, and then use these SDs to produce ODTs. Due to its physicochemical properties, FF is regarded as challenging for drug dissolution improvement, and the incorporation into a mesoporous silica material has been proven successful for it in the previously published studies [15,16,17]. Since the aim was to transform FF from crystalline to amorphous state, pure SDs as well as ODTs should have improved dissolution rate compared to crystalline FF or physical mixtures of FF and co-processed excipient.

## 2. Materials and Methods

### 2.1. Materials

Mesoporous silica (Syloid^®^ 244 FP) was obtained from Grace Davison, Grace GmbH & Co. KG (Worms, Germany), and isomalt (GalenIQ^®^ 800) was obtained from Beneo (Mannheim, Germany). FF was obtained from Biosynth^®^, Carbosynth (Berkshire, UK). Magnesium stearate and mannitol (Parteck ^®^ M) were obtained from Merck KGaA (Darmstadt, Germany). Sodium croscarmellose (Ac-Di-Sol^®^) was obtained from IFF’s Pharma Solutions (New York, NY, USA). Sorbitol (Neosorb^®^ P 300 DC) was obtained from Roquette (Lestrem, France). Low-substituted hydroxypropylcellulose (L-HPC) was obtained from Shin-Etsu Chemical Co., Ltd. (Tokyo, Japan). All other materials used in the study were of reagent grade. Water was purified by reverse osmosis.

### 2.2. Methods

#### 2.2.1. Preparation of a Co-Processed Excipient

Preparation and characterization of a co-processed material, comprising of mesoporous silica and isomalt, are described in detail in our previous work [4]. Briefly, high-shear wet granulation was used for its production, and aDesign of Experiments approach was used to investigate which process parameters have a significant influence on the properties of the co-processed material. After a thorough analysis, an optimization experiment was performed in order to develop a material with optimal characteristics for drug loading and compression into tablets—i.e., high specific surface area (141 m^2^/g) and appropriate particle size (380 µm) for suitable compression properties. This is the product that was used in the present study.

#### 2.2.2. Preparation of SDs

SDs with FF and a co-processed excipient were prepared by solvent evaporation method in a rotary evaporator (IKA RV 05, Staufen, Germany). SDs (8 g) with 20%, 30% and 40% theoretical FF content were prepared by dissolving the corresponding amount of FF in 100 mL of ethyl acetate, followed by the addition of a co-processed excipient. The choice of ethyl acetate as a solvent was based on a previous study, where it gave the best resulting SDs of FF and pure mesoporous silica out of three tested solvents [18]. The suspensions obtained were evaporated in a rotary evaporator at 50 rpm at the boiling point temperature of ethyl acetate, which is 77 °C at normal air pressure [19]. This process lasted up to 30 min. After solvent was not visible in the flask anymore, the pressure was reduced to <10 mbar for 30 min to ensure complete solvent removal. Physical mixtures (PMs) with 20%, 30% and 40% FF were prepared by mixing the corresponding amounts of a co-processed excipient and FF for approximately 5 min with 3D motion mixer (Inversina, Bioengineering, Wald, Switzerland).

#### 2.2.3. Characterization of SDs

DSC analysis was carried out on each newly prepared SD to ascertain the solid-state properties of FF. The experiments were performed using the DSC1 STARe system (Mettler Toledo, Columbus, OH, USA). Approximately 5–10 mg of each sample was heated in an aluminum pan with a perforated lid, from 0 °C to 110 °C, at a heating rate of 10 °C/min under a nitrogen gas flow of 50 mL/min. An empty aluminum pan served as the reference. Data obtained were analyzed using the STARe V9.30 software.

The FF content in the SDs was quantified using UV spectroscopy, with absorbance measured at 290 nm. Precisely weighed SD samples were suspended in acetone to extract FF. After ultrasonicating for 10 min, the samples were filtered through a 0.45 µm pore RC membrane filter to eliminate particles of the co-processed excipient and adequately diluted before measuring the absorbance. The FF concentration was determined using previously established calibration curves of FF in acetone. The actual FF content in the SDs was expressed as a percentage of the theoretical content.

Drug release studies were conducted using a USP II apparatus with rotating paddles (VanKel VK 7010 Tablet Dissolution Tester, VanKel Technology Group, Cary, NC, USA). Samples of SDs and physical PMs containing 150 mg of FF were tested in triplicates and depicted as the cumulative percentage of FF released over time. They were placed in 900 mL of the selected discriminatory dissolution medium at 37.5 °C ± 1 °C (0.1 M HCl, pH 1.2, 0.01 M SDS, 0.034 M NaCl). At specified intervals (5, 10, 15, 30, 45, 60, 90 and 120 min), 5 mL of the medium was withdrawn and filtered through a 0.45 µm pore RC membrane filter. The medium was not replenished. The samples were subsequently analyzed using UV spectroscopy, with absorbance measured at 290 nm. The FF concentration was determined using previously established calibration curves of FF in the dissolution medium.

To assess the similarity between dissolution profiles, similarity factor *f*_2_ proposed by Moore and Flanner was determined for the relevant pair of formulations. This is a widely used and model-independent approach used to mathematically assess whether two dissolution profiles are similar [20]. It is calculated by Equation (1):

(1) $$f_{2}=50*\log\left\{{\left[1+\frac{1}{n}\sum_{j=1}^{n}{\left|R_{j}-T_{j}\right|}^{2}\right]}^{-0.5}*100\right\},$$

where *n* is the number of time points at which the sample was withdrawn, *R_j_* is the percentage dissolved at time point *j* for reference formulation, and *T_j_* is the percentage dissolved at the same time point for test formulation. The value *f*_2_ lies between 0 and 100, and a value larger than 50 indicates similarity, while a value below 50 indicates dissimilarity [21].

#### 2.2.4. Tableting

ODTs were prepared from the 30% SD using a single-punch tablet press (Killian SP300, IMA, Cologne, Germany) with convex-faced punches (2r = 12 mm). Several tableting mixtures were compressed, and the masses of ingredients as well as their roles in the formulations (F1–F5) are given in Table 1.

The tablets contained a targeted amount of 150 mg of FF, which corresponds to a single therapeutic dose. All ingredients except for magnesium stearate were mixed for 3 min by a 3D motion mixer (Inversina, Bioengineering, Wald, Switzerland), followed by the addition of magnesium stearate and another 1 min of mixing in the same manner. Tablets were compressed at tableting speed 15 punches per minute, and with the compression force 6.5–7.5 kN.

The drug release experiments from ODTs were conducted similarly to those for SDs in powder form. Drug release from the tablets was compared to drug release from 30% SD, 30% PM and pure crystalline FF. 

Disintegration time (Erweka ZT4, Erweka GmbH, Langen, Germany) and tablet hardness (Kraemer HC6.2, Kraemer Elektronik GmbH, Darmstadt, Germany) were determined according to the monographs from European Pharmacopeia 10th edition [22]. Disintegration time was evaluated on 3 tablets and tablet hardness on 5 tablets.

## 3. Results and Discussion

In the first part of the study, SDs with three different FF ratios were prepared by the rotary evaporation method, and the changes in FF solid state and dissolution were investigated in comparison to PMs with the same FF ratios and pure crystalline FF. The measured FF content in all prepared SDs ranged from 91.4% to 96.8%, indicating that the incorporation of FF into silica particles was successful. In the second part of the study, the SD, which was identified as the best, was used to prepare several formulations for the compression of ODTs. Our aim was to produce a formulation which would have a comparable drug release profile before and after the compression.

### 3.1. Drug Release from SDs and PMs

**Figure 2:**
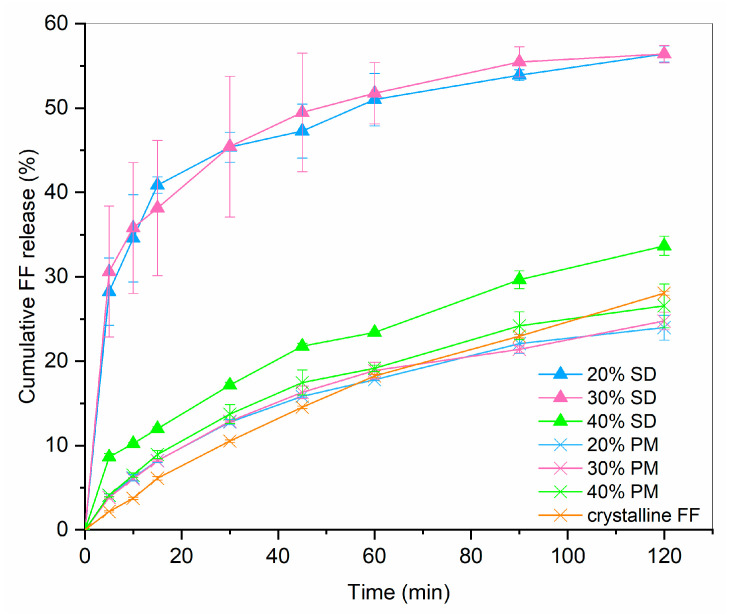
FF release profile from SDs and PMs in comparison to dissolution of crystalline FF (*n* = 3).

**Figure 3:**
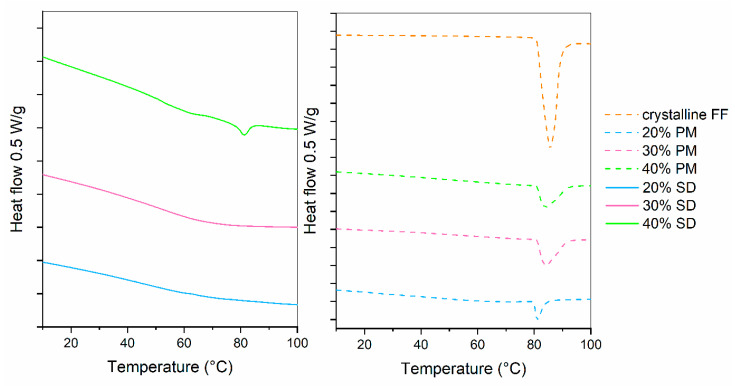
DSC curves of SDs, PMs and crystalline FF.

Drug release profiles of 20%, 30% and 40% SDs and PMs are shown in Figure 2. For comparison, the dissolution of crystalline FF is also given. It can be seen that drug release from PMs hardly differs from pure crystalline FF, which is not surprising considering that the results from DSC showed a large portion of crystalline FF in all PMs (see Figure 2; fusion enthalpies for 20%, 30% and 40% FF are 7.7 J/g, 17.2 J/g and 23.1 J/g, respectively). On the other hand, 20% and 30% SDs both exhibit a significantly faster dissolution (4.5-fold higher after 30 min than pure crystalline FF) and more than two-fold higher drug release after 2 h. This is most likely due to the conversion of FF from crystalline to amorphous state, which was confirmed by the DSC measurements (see Figure 3). However, the 40% SD exhibits a significantly slower drug release, likely attributed to a substantial presence of crystalline FF within the sample. In fact, the *f*_2_ similarity factor between 40% SD and crystalline FF is 59.8, meaning that the profiles are considered similar. This crystallinity can be discerned by the occurrence of two minor endothermic peaks in the DSC curves (fusion enthalpy of both peaks combined is below 4 J/g). Although higher drug loading is generally desired, since it can importantly influence the size of a final dosage form, this should not be at the expanse of some API remaining in crystalline state. Not only is the drug release from such a formulation slower, but its stability can also be compromised because of the already existing crystallization nuclei. That is why the 30% SD was chosen as the best candidate for further experimentation to produce ODTs.

### 3.2. Production and Characterization of ODTs

**Figure 4:**
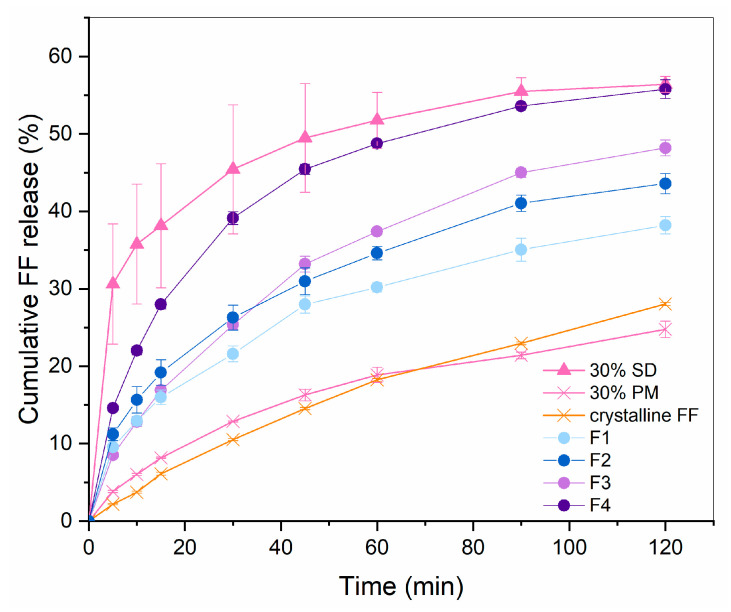
FF release profile from F1–F4 in comparison to 30% SD, 30% PM and crystalline FF (*n* = 3).

In the preliminary studies, it was observed that capping, lamination and friability around the edges are likely to occur at compression of the developed co-processed material, which was especially problematic at higher tableting speed and using flat-faced punches. To reduce the risk of these issues, a low tableting speed (15 tablets per minute) and convex-shaped punches were used for the compression of the prepared formulations F1–F5. The results of disintegration and hardness testing are given in Table 2. For higher clarity, amounts of mannitol/sorbitol as wicking agents and direct compression excipients, LHPC and sodium croscarmellose are listed again. Figure 4 displays the drug release profiles for all the successfully compressed formulations.

F1 and F2 consist of mannitol as a wicking agent, selected for its frequent use in ODTs due to its pleasant taste, cooling sensation, rapid dissolution and low hygroscopicity [23,24]. In our study, however, its use required the addition of L-HPC to prevent tablet capping and lamination, owing to a high immediate elastic recovery of this excipient after the axial pressure of the upper tablet punch is removed [25,26]. However, this resulted in a higher tablet mass to ensure the right amount FF, which certainly presents a disadvantage from the patients’ point of view. It can be seen that the disintegration time of tablets made with F1 and F2 is under 1 min, which well meets the requirement of Ph. Eur. that ODTs should disintegrate in 3 min [22]. However, the rapid disintegration observed in the formulation is not congruent with the dissolution rate, which remains significantly lower than that of pure SD. This is most likely due to the presence of L-HPC, which can also be used for sustained drug release in some formulations due to its swelling properties in an aqueous environment [27]. Notably, the dissolution rate comparison between formulations F1 and F2 reveals an inverse relationship with the L-HPC content: the higher the L-HPC content, the lower the dissolution rate, although the difference in it was not spotted by the calculated similarity factor 68.7.

Since ODTs made with mannitol required the addition of L-HPC, which resulted in insufficient drug dissolution rate, it was replaced with another sugar alcohol which can also be used in orodispersible formulations, namely sorbitol. Although it is more hygroscopic, it is better compressible than mannitol, needs no addition of LHPC and hence, it was recognized as more appropriate for our formulations [27]. The disintegration times of F3 and F4 were somewhat higher than of F1 and F2, but still well below the threshold of 3 min. However, it has to be noted that this threshold is quite high from the patients’ perspective, which is why the American Food and Drug Administration (FDA) gave more strict recommendations [28]. According to the document published in 2008, the desired disintegration time should be under 30 s, which has not been achieved in any of our formulations.

Drug release after two hours from F3 and F4 was significantly higher than from F1 and F2, which shows that the formulations with sorbitol are indeed more feasible in our case. In fact, the amount of released FF from F4 is almost the same as from pure 30% SD (similarity factor 52.7 which shows similarity), which is in line with the set aim. The initial release rate was still slower than before the compression, which might have been solved with the addition of even more superdissintegrant, assumed from the fact that F4 with a higher content of sodium croscarmellose shows much faster dissolution rate than F3, which has a lower sodium croscarmellose content. However, this would result in a higher tablet mass, which is an important drawback considering that these are meant to be patient-friendly dosage forms. In fact, the FDA recommendations mentioned earlier also suggest the tablet weight to be 500 mg or less. Since the weight of F3 and F4 tablets is larger than 600 mg already, it was decided that achieving an even faster initial FF release at the expanse of a larger tablet size would be unfeasible. In a recent research study published by Kovačević et al. [29], which also dealt with producing ODTs based on Syloid 244 FP granules, the tablet weight of the optimized formulation was even larger than 800 mg, despite the lower required dose of the API (carvedilol; 12.5 mg). This indicates that Syloid is indeed a challenging excipient for the production of ODTs and that further research is needed to fully understand its potential for such use. 

With the aim of producing a smaller tablet, we tried to compress tablets from F5, which contained equal amounts of sodium croscarmellose and sorbitol. However, the tablets could not be compressed due to severe lamination. Apparently, the amount of ingredients other than the SD in this case is not sufficient to prevent this issue, which has been recognized as a downside of our developed co-processed material, as mentioned earlier.

## 4. Conclusions

The preparation of SDs with a co-processed material and the poorly water-soluble model drug FF successfully improved its dissolution in a discriminatory medium. Changes in the solid state of the API during the rotary evaporation process were confirmed, resulting in better dissolution performance than simple physical mixing of FF and the excipient. SD containing 30% FF showed no signs of crystallinity and significantly enhanced dissolution. This SD was successfully formulated into ODTs with small amounts of additional excipients. The drug release profile of the ODTs closely matched that of the uncompressed SD, although some differences remained, even with the optimal tablet formulation. To further enhance patient acceptability, future efforts should focus on reducing the disintegration time and tablet weight/size. Furthermore, tablet characterization tests should be performed on a greater number of tablets and formulations, which can be viewed as a limitation of our study. Another limitation of our study is a lack of in vivo pharmacokinetic studies, which would give even more solid evidence concerning the relevance of our results in vitro. Despite the challenges posed by the co-processed excipient and the SDs prepared from it, this study provides a solid foundation and potential for further exploration of this approach, even in terms of scale-up and production. 

## Figures and Tables

**Table 1:** Composition of the tableting mixtures; ingredients, their role in the mixture and their exact mass per one tablet.

Ingredient		Role	Amount per Tablet (mg)				
			F1	F2	F3	F4	F5
**30% SD**	**FF**	API (active pharmaceutical ingredient)	150	150	150	150	150
	**Co-processed excipient**	Carrier	350	350	350	350	350
**Sodium crosscarmellose**		Superdissintegrant	50	50	50	75	50
**Sorbitol**		Wicking agent, direct compression excipient	0	0	75	50	50
**Mannitol**		Wicking agent, direct compression excipient	75	75	0	0	0
**L-HPC**		Anti-capping agent	50	31.3	0	0	0
**Magnesium stearate**		Glidant	6.8	6.6	6.3	6.3	6.0
**Total tablet mass (mg)**			**681.8**	**662.9**	**631.3**	**631.3**	**606.0**

**Table 2:** Disintegration time and hardness of tablets made of F1–F5.

Formulation	Amount of Mannitol per Tablet (mg)	Amount of Sorbitol per Tablet (mg)	Amount of L-HPC per Tablet (mg)	Amount of Sodium Croscarmellose per Tablet (mg)	Disintegration Time (*n* = 3)	Hardness (*n* = 5; Average ± Standard Deviation)
**F1**	75	-	50	50	<60 s	38 N ± 3 N
**F2**	75	-	31.3	50	<60 s	41 N ± 3 N
**F3**	-	75	-	50	≈90 s	34 N ± 5 N
**F4**	-	50	-	75	<80 s	29 N ± 4 N
**F5**	-	50	-	50	Tablets could not be produced due to capping and lamination	

## Data Availability

Data is contained within the article.
